# Supplementary material for: A Bayesian psychophysics model of sense of agency
Source: Nat Commun. 2019 Sep 18;10:4250. doi: 10.1038/s41467-019-12170-0 (PMC6751175; doi:10.1038/s41467-019-12170-0)
Supplement: Supplementary file 1 — Supplementary Source Codes [file 41467_2019_12170_MOESM1_ESM.pdf]

**Supplementary Source Codes**

For the manuscript "A Bayesian psychophysics model of sense of agency"

by Roberto Legaspi & Taro Toyoizumi
Emails: {roberto.legaspi, taro.toyoizumi}@riken.jp

This supplementary information contains the MATLAB source codes that were used to generate the simulation data and plot the computation results for analyses.

Following is the order in which the main codes should be compiled and executed:

1. create_SimulationData.m.
2. find_muAO.m
3. compute_PXisPrcShfts.m. The plots that were generated here were shown in Figs. 2 and 5b.
4. compute_PerTrialPrcShfts.m. The plots were shown in Figs. 3b, 3c, 3d and 3e, as well as 4c and 4d.
5. compute_CCEPXi1.m. The plots that were generated from this were shown in Figs. 4a and 6a.
6. compute_PerTrialCCE.m. The plots were shown in Figs. 4b and 6b.

The descriptions and objectives of each of the above are written in their codes. Note that to generate the results that correspond to the two intentional binding experiments that were studied, the variable *Expr* should be assigned the value 1 and 2 for Haggard et al.'s (Ref [3]) and Wolpe et al.'s (Ref [22]) studies, respectively.

Lastly, the following auxiliary source codes are called by the main codes described above:

1. soa_IBexperiment.m
2. soa_IBTargets.m
3. soa_InitMatrix.m
4. soa_sortMatrices.m
5. soa_plotPrcShfts.m
6. soa_plotErrorBars.m
7. soa_plotBehaviors.m

```
% Published: August 14, 2019
% Copyright
%   Lab for Neural Computation and Adaptation
%   RIKEN Center for Brain Science
%
% Objective: Create the noisy sensory input signals arriving at various times taoA and taoO

% Cleaners
clear                                                                  % clears all variables from the workspace
clc                                                                    % clears the command window
close all                                                              % closes all figures (such as plots)

%{
Only if random generation should be repeatable
rng(1,'twister');
%}

% Simulation conditions
taoInstances = 35000;                                                  % Number of taoA and taoO instances to be generated
ExpR = 1; numCond = 3;                                                 % Experimental set-up
                                                                       %   Haggard et al. (2002): ExpR = 1; NumCond = 3; (Voluntary, Involuntary, Sham)
                                                                       %   Wolpe et al. (2013)  : ExpR = 2; NumCond = 3; (Low, Intermediate, High)
tAp=0; dist_tAtO=250; tOp=tAp+dist_tAtO;                               % Actual physical stimulus timings

for CondBO = 1:numCond
    % Initialize baseline parameters with reported empirical data
    [muA, sigmaA, muO, sigmaO] = soa_IBexperiment(ExpR, CondBO);

    % Generate from Gaussian distributions
    Vec_taoA = normrnd(tAp + muA, sigmaA, [1 taoInstances]);           % Alterntaive: sigmaA .* randn(1,numInstances) + (tAp + muA);
    Vec_taoO = normrnd(tOp + muO, sigmaO, [1 taoInstances]);           %              sigmaO .* randn(1,numInstances) + (tOp + muO);

    %{
    % Check the generated data
    figure; normplot(Vec_taoA);
    figure; normplot(Vec_taoO);
    figure; histfit(Vec_taoA);
    figure; histfit(Vec_taoO);
    %}

    % Generate statistics
    sizeVec_taoA = numel(Vec_taoA);
    sizeVec_taoO = numel(Vec_taoO);
    taoA_min = min(Vec_taoA); taoA_max = max(Vec_taoA);
    taoO_min = min(Vec_taoO); taoO_max = max(Vec_taoO);
    uVectaoA = mean(Vec_taoA); stdVectaoA = std(Vec_taoA);
    uVectaoO = mean(Vec_taoO); stdVectaoO = std(Vec_taoO);

    % Store generated simulation data
    fprintf('\n============== tao DataSet Exp %d Cond %d ================\n', ExpR, CondBO);
    fprintf('taoA [%0.2f, %0.2f] taoO [%0.2f, %0.2f]\n', taoA_min,taoA_max, taoO_min,taoO_max);
    fprintf('tao statistics: %0.2f (%0.2f)\t %0.2f (%0.2f)\n', uVectaoA, stdVectaoA, uVectaoO, stdVectaoO);
    fprintf('taoA elements: %d taoO elements: %d\n', sizeVec_taoA, sizeVec_taoO);
    fprintf('============================================\n');
    fnametaoA = sprintf('Exp%dCond%d_Vec_taoA.csv',ExpR,CondBO);
    fnametaoO = sprintf('Exp%dCond%d_Vec_taoO.csv',ExpR,CondBO);
    dlmwrite(fnametaoA, Vec_taoA,'delimiter',',');
    dlmwrite(fnametaoO, Vec_taoO,'delimiter',',');
end
```

```
% Published: August 14, 2019
% Copyright
%   Lab for Neural Computation and Adaptation
%   RIKEN Center for Brain Science
%
% Objective: Find the optimal value for the free parameter muAO using Haggard et al.'s data,
%               with sigmaAO equal to 10ms.

% Cleaners
clear                                                                   % clears all variables from the workspace
clc                                                                     % clears the command window
close all                                                               % closes all figures (such as plots)

% Simulation Conditions
taoInstances = 35000;                                                   % Number of taoA and taoO instances to be generated
ExpR = 1; numCond = 3;                                                  % Experimental set-up
                                                                        %   Haggard et al. (2002): ExpR = 1; NumCond = 3; (Vol, Invol, Sham)
tAp=0; dist_tAtO=250; tOp=tAp+dist_tAtO;                                % Actual physical stimulus timings

sigmaAO = 10;                                                           % To obtain discernible perceptual shifts, sigmaAO should be small

for muAO = [190 200 210 220 230 240 250]

    fprintf('Action and outcome perceptual shifts per condition given muAO=%d\n', muAO);
    sumError = 0;

    for CondBO = 1:numCond

        % Read from files taoA and taoO values derived from a Gaussian distribution
        fnametaoA = sprintf('Exp%dCond%d_Vec_taoA.csv',ExpR,CondBO);
        fnametaoO = sprintf('Exp%dCond%d_Vec_taoO.csv',ExpR,CondBO);
        Vec_taoA  = dlmread(fnametaoA);
        Vec_taoO  = dlmread(fnametaoO);

        % Get the reported empricial baseline parameters
        [muA, sigmaA, muO, sigmaO] = soa_IBexperiment(ExpR, CondBO);

        % Compute for sigma_Tot
        sigmaTot2 = sigmaA^2 + sigmaO^2 + sigmaAO^2;

        % Compute the action and oputcome perceptual shifts
        Vec_PrcShftA   =   (sigmaA^2 / sigmaTot2) * (Vec_taoO - Vec_taoA - muAO);
        Vec_PrcShftO   = - (sigmaO^2 / sigmaTot2) * (Vec_taoO - Vec_taoA - muAO);
        uVec_PrcShftA  = mean(Vec_PrcShftA); sdVec_PrcShftA = std(Vec_PrcShftA);
        uVec_PrcShftO  = mean(Vec_PrcShftO); sdVec_PrcShftO = std(Vec_PrcShftO);
        ruVec_PrcShftA = round(uVec_PrcShftA); rsdVec_PrcShftA = round(sdVec_PrcShftA);
        ruVec_PrcShftO = round(uVec_PrcShftO); rsdVec_PrcShftO = round(sdVec_PrcShftO);

        % Compute for model estimation errors given the reported empirical results
        [targPrcShftA, targPrcShftO] = soa_IBTargets(ExpR,CondBO);
        errPrcShft = abs(ruVec_PrcShftA - targPrcShftA);
            %{
                NOTE: Even when we consider the average of action and outcome estimation errors,
                        the optimal result is the same.
                errPrcShft = (abs(ruVec_PrcShftA - targPrcShftA) + abs(ruVec_PrcShftO - targPrcShftO))/2;
            %}
        sumError = sumError + errPrcShft;

        fprintf('Condition %d:\t %0.1f(%0.2f)\t %0.1f(%0.2f)\n', CondBO, ruVec_PrcShftA, rsdVec_PrcShftA, ruVec_PrcShftO, rsdVec_PrcShftO);
    end

    modelEE = sumError/numCond;
    fprintf('model estimation error:\t%0.2f:\n\n',modelEE);
    if muAO==190 || (muAO~=190 && modelEE < min_modelEE)
```

```
        min_modelEE = modelEE;
        opt_muAO = muAO;
    end
end

fprintf('Optimal muAO is %d ms.\n', opt_muAO);

%{
    Notes to METHODS:
    - Estimates of the perceptual shift in action timing alone was sufficient to indicate
        the optimal muAO.
    - The optimal muAO for Experiment 1 (Haggard et al.) is 230 ms.
    - Retain this same muAO value for Experiment 2 (Wolpe et al.).
 %}
```

```
% Published: August 14, 2019
% Copyright
%   Lab for Neural Computation and Adaptation
%   RIKEN Center for Brain Science
%
% Objective: Fit Haggard et al's data to find the optimal value for the free parameter P(Xi=1),
%               muAO is 230ms and sigmaAO is 10ms.
%            Plot the action and outcome perceptual shifts given P(Xi=1) in the range
%               [0.0,1.0] with 0.1 increments

% Cleaners
clear                                                                       % clears all variables from the workspace
clc                                                                         % clears the command window
close all                                                                   % closes all figures (such as plots)

% Graph display fonts
fontsize = 20;
sizeBin  = 200;

% Simulation Conditions
taoInstances = 35000;                                                       % Number of taoA and taoO instances to be generated
ExpR = 1; numCond = 3;                                                      % Experimental set-up
                                                                            %   Haggard et al. (2002): ExpR = 1; NumCond = 3; (Vol, Invol, Sham)
                                                                            %   Wolpe et al. (2013)  : ExpR = 2; NumCond = 3; (Low, Int, High)
tAp=0; dist_tAtO=250; tOp=tAp+dist_tAtO;                                    % Actual physical stimulus timings

% Optimal condition-independent parameters
muAO    = 230;
sigmaAO = 10;

% Interval length in consideration
T = 250;                                                                    % Large enough but finite constant

% Data Matrices
LB = 0.0; INC = 0.1; UB = 1.0;
arrPXi1     = LB:INC:UB;
size_pXi1   = numel(arrPXi1);
arrPrcShftA = zeros(numCond,size_pXi1);
arrPrcShftO = zeros(numCond,size_pXi1);
arrAOBinding = zeros(numCond,size_pXi1);

for CondBO = 1:numCond

    % Read from files taoA and taoO values derived from a Gaussian distribution
    fnametaoA = sprintf('Exp%dCond%d_Vec_taoA.csv',ExpR,CondBO);
    fnametaoO = sprintf('Exp%dCond%d_Vec_taoO.csv',ExpR,CondBO);
    Vec_taoA = dlmread(fnametaoA);
    Vec_taoO = dlmread(fnametaoO);

    indxPXi1 = size_pXi1 + 1;
    for PXi_1 = UB:-INC:LB
        PXi_0 = 1 - PXi_1;

        % Matrices to track optimal action and outcome estimates
        Vec_PrcShftA  = soa_InitMatrix(1,taoInstances);
        Vec_PrcShftO  = soa_InitMatrix(1,taoInstances);
        Vec_AOBinding = soa_InitMatrix(1,taoInstances);

        for indx_tao = 1:taoInstances

            % Do for each pair of taoA and taoO
            taoA = Vec_taoA(indx_tao);
            taoO = Vec_taoO(indx_tao);
```

```
            % Get the reported empricial baseline parameters
            [muA, sigmaA, muO, sigmaO] = soa_IBexperiment(ExpR, CondBO);

            % Compute for the posterior-ratio
            Z1 = sqrt(2*pi)*sigmaAO*T;
            Z0 = T^2;
            Theta = log((PXi_1*Z0)/(PXi_0*Z1));
            sigmaTot2 = sigmaA^2 + sigmaO^2 + sigmaAO^2;
            r = exp(Theta - ((taoO-taoA-muAO)^2/(2*sigmaTot2)));

            % Compute for strength of temporal binding
            if r > 1 % Causal
                tAhat = taoA + (sigmaA^2/sigmaTot2)*(taoO-taoA-muAO);
                tOhat = taoO - (sigmaO^2/sigmaTot2)*(taoO-taoA-muAO);
                Xihat =1;
            else % Acausal
                tAhat = taoA;
                tOhat = taoO;
                Xihat=0;
            end

            Vec_PrcShftA(1, indx_tao)  = tAhat - taoA;
            Vec_PrcShftO(1, indx_tao)  = tOhat - taoO;
            Vec_AOBinding(1, indx_tao) = 250 + (tOhat-taoO) - (tAhat-taoA);
        end

        uPrcShftA  = mean(Vec_PrcShftA(:));  sdPrcShftA  = std(Vec_PrcShftA(:));
        uPrcShftO  = mean(Vec_PrcShftO(:));  sdPrcShftO  = std(Vec_PrcShftO(:));
        uAOBinding = mean(Vec_AOBinding(:)); sdAOBinding = std(Vec_AOBinding(:));

        % Compute for model estimation errors given the reported empirical results
        [targPrcShftA, targPrcShftO] = soa_IBTargets(ExpR,CondBO);

        ruVec_PrcShftA = round(uPrcShftA);
        ruVec_PrcShftO = round(uPrcShftO);
        errPrcShftA = abs(ruVec_PrcShftA - targPrcShftA);
        errPrcShftO = abs(ruVec_PrcShftO - targPrcShftO);

        fprintf('Condition %d  \t P(Xi=1): %0.2f\n', CondBO, PXi_1);
        fprintf('uPercShfts   :\t %0.2f(%0.2f)\t %0.1f(%0.1f)\n', uPrcShftA, sdPrcShftA, uPrcShftO,sdPrcShftO);
        fprintf('Error in action  perceptual shift: %0.2f\n', errPrcShftA);
        fprintf('Error in outcome perceptual shift: %0.2f\n\n', errPrcShftO);

        indxPXi1 = indxPXi1 - 1;

        arrPrcShftA(CondBO,indxPXi1)  = uPrcShftA;
        arrPrcShftO(CondBO,indxPXi1)  = uPrcShftO;
        arrAOBinding(CondBO,indxPXi1) = uAOBinding;
    end
    fprintf('\n');

end

% Plot and store the perceptual shifts and action-outcome binding

soa_plotPrcShts(ExpR, arrPrcShftA, arrPrcShftO, arrPXi1, fontsize);
fnamePrcShft = sprintf('Exp%d_PXisPrcShfts.png',ExpR);
saveas(gcf,fnamePrcShft);

soa_plotBehaviors(ExpR, arrAOBinding,  arrPXi1, fontsize, 1);
fnamePrcShft = sprintf('Exp%d_PXisPrcShfts.png',ExpR);
saveas(gcf,fnamePrcShft);

% Store the perceptual shifts
```

```
fnamePrcShftA = sprintf('Exp%d_arrPrcShftA.csv',ExpR);
fnamePrcShftO = sprintf('Exp%d_arrPrcShftO.csv',ExpR);
dlmwrite(fnamePrcShftA, arrPrcShftA,'delimiter',',');
dlmwrite(fnamePrcShftO, arrPrcShftO,'delimiter',',');

%{
    Notes to METHODS:
    - Estimates of the perceptual shift in action timing alone was sufficient to indicate
        the optimal P(Xi=1) value. However, note the following.
    - Although the optimal P(Xi=1) value for the voluntary and involuntary conditions is 1.0,
        the result is saturated. Hence, report P(Xi=1)=0.9 for both conditions.
    - Report P(Xi=1)=0.1 for the sham condition, assuming causality is less frequently detected.
    - Although the ptimal P(Xi=1) value for the intermediate tone uncertainty condition is 0.5,
        the outcome binding behavior is not consistent with the reported outcome binding.
        Report P(Xi=1)=0.6 for the intermediate tone uncertainty condition since it also best minimized
        the estimation error while reproducing the reported action and outcome bindings.
 %}
```

```
% Published: August 14, 2019
% Copyright
%   Lab for Neural Computation and Adaptation
%   RIKEN Center for Brain Science
%
% Objective: Compute the trial-to-trial temporal binding and repulsion effects,
%               as well as the baseline and operant temporal bindings,
%               as functions of temporal disparity, i.e., taoO-taoA

% Cleaners
clear                                                                   % clears all variables from the Workspace
clc                                                                     % clears the Command Window
close all

% Graph display fonts
fontsize = 20;
sizeBin  = 200;

% Simulation Conditions
taoInstances = 35000;                                                   % Number of taoA and taoO instances to be generated
ExpR = 2; numCond = 3;                                                  % Experimental set-up
                                                                        %   Haggard et al. (2002): ExpR = 1; NumCond = 3; (Vol, Invol, Sham)
                                                                        %   Wolpe et al. (2013)  : ExpR = 2; NumCond = 3; (Low, Int, High)
tAp=0; dist_tAtO=250; tOp=tAp+dist_tAtO;                                % Actual physical stimulus timings

% Optimal condition-independent parameters
muAO    = 230;
sigmaAO = 10;

% Interval length in consideration
T = 250;                                                                % Large enough but finite constant

% Data Matrices
Vec_PrcShftA   = zeros(numCond,taoInstances);
Vec_PrcShftO   = zeros(numCond,taoInstances);
Vec_taoI       = zeros(numCond,taoInstances);
Vec_OpPrcShfts = zeros(numCond,taoInstances);
Vec_BsPrcShfts = zeros(numCond,taoInstances);

for CondBO = 1:numCond

    % Read from files taoA and taoO values derived from a Gaussian distribution
    fnametaoA = sprintf('Exp%dCond%d_Vec_taoA.csv',ExpR,CondBO);
    fnametaoO = sprintf('Exp%dCond%d_Vec_taoO.csv',ExpR,CondBO);
    Vec_taoA = dlmread(fnametaoA);
    Vec_taoO = dlmread(fnametaoO);

    % Simulated using the fitted P(Xi=1) optimal values
    if ExpR==1
        if CondBO == 1
            PXi_1 = 0.9;
        elseif CondBO == 2
            PXi_1 = 0.9;
        else
            PXi_1 = 0.1;
        end
    elseif ExpR==2
        if CondBO == 1
            PXi_1 = 0.9;
        elseif CondBO == 2
            PXi_1 = 0.6;
        else PXi_1 = 0.5;
        end
    end
```

```matlab
    PXi_0 = 1 - PXi_1;

    for indx_tao = 1:taoInstances

        % Do for each pair of taoA and taoO
        taoA = Vec_taoA(indx_tao);
        taoO = Vec_taoO(indx_tao);

        % Get the reported empricial baseline parameters
        [muA, sigmaA, muO, sigmaO] = soa_IBexperiment(ExpR, CondBO);

        % Compute for the posterior-ratio
        Z1 = sqrt(2*pi)*sigmaAO*T;
        Z0 = T^2;
        Theta = log((PXi_1*Z0)/(PXi_0*Z1));
        sigmaTot2 = sigmaA^2 + sigmaO^2 + sigmaAO^2;
        r = exp(Theta - ((taoO-taoA-muAO)^2/(2*sigmaTot2)));

        % Compute for strength of temporal binding
        if r > 1 % Causal
            tAhat = taoA + (sigmaA^2/sigmaTot2)*(taoO-taoA-muAO);
            tOhat = taoO - (sigmaO^2/sigmaTot2)*(taoO-taoA-muAO);
            Xihat =1;
        else % Acausal
            tAhat = taoA;
            tOhat = taoO;
            Xihat=0;
        end

        Vec_PrcShftA(CondBO, indx_tao)     = tAhat - taoA;
        Vec_PrcShftO(CondBO, indx_tao)     = tOhat - taoO;
        Vec_BsPrcShfts(CondBO, indx_tao)   = taoO  - taoA;
        Vec_OpPrcShfts(CondBO, indx_tao)   = tOhat - tAhat;
        Vec_taoI(CondBO, indx_tao)         = taoO  - taoA;
    end
end

% Plot and store the perceptual shifts

sortedtaoI = Vec_taoI;
[sortedtaoI(1,:), sortIndx1] = sort(Vec_taoI(1,:));
[sortedtaoI(2,:), sortIndx2] = sort(Vec_taoI(2,:));
[sortedtaoI(3,:), sortIndx3] = sort(Vec_taoI(3,:));
sortedPrcShftA   = soa_sortMatrices(Vec_PrcShftA,   sortIndx1, sortIndx2, sortIndx3);
sortedPrcShftO   = soa_sortMatrices(Vec_PrcShftO,   sortIndx1, sortIndx2, sortIndx3);
sortedOpPrcShfts = soa_sortMatrices(Vec_OpPrcShfts, sortIndx1, sortIndx2, sortIndx3);
sortedBsPrcShfts = soa_sortMatrices(Vec_BsPrcShfts, sortIndx1, sortIndx2, sortIndx3);

soa_plotErrorBars(ExpR, sortedtaoI, sortedPrcShftA, fontsize, 1, sizeBin);
fnamePrcShft = sprintf('Exp%d_perTrialPrcShftA.png',ExpR);
saveas(gcf,fnamePrcShft);
soa_plotErrorBars(ExpR, sortedtaoI, sortedPrcShftO, fontsize, 1, sizeBin);
fnamePrcShft = sprintf('Exp%d_perTrialPrcShftO.png',ExpR);
saveas(gcf,fnamePrcShft);
soa_plotErrorBars(ExpR, sortedtaoI, sortedBsPrcShfts, fontsize, 1, sizeBin);
fnamePrcShft = sprintf('Exp%d_perTrialBaselinePrcShfts.png',ExpR);
saveas(gcf,fnamePrcShft);
soa_plotErrorBars(ExpR, sortedtaoI, sortedOpPrcShfts, fontsize, 1, sizeBin);
fnamePrcShft = sprintf('Exp%d_perTrialOperantPrcShfts.png',ExpR);
saveas(gcf,fnamePrcShft);
```

```
% Published: August 14, 2019
% Copyright
%   Lab for Neural Computation and Adaptation
%   RIKEN Center for Brain Science
%
% Objective: Compute the Confidence on Causal Estimate (CCE) along P(Xi=1)
%                values in the range [0.0,1.0] with increments of 0.1

% Cleaners
clear                                                                          % clears all variables from the Workspace
clc                                                                            % clears the Command Window
close all

% Graph display fonts
fontsize = 20;
sizeBin  = 200;

% Simulation Conditions
taoInstances = 35000;                                                          % Number of taoA and taoO instances to be generated
ExpR = 2; numCond = 3;                                                         % Experimental set-up
                                                                               %   Haggard et al. (2002): ExpR = 1; NumCond = 3; (Vol, Invol, Sham)
                                                                               %   Wolpe et al. (2013)  : ExpR = 2; NumCond = 3; (Low, Int, High)
tAp=0; dist_tAtO=250; tOp=tAp+dist_tAtO;                                      % Actual physical stimulus timings

% Optimal condition-independent parameters
muAO    = 230;
sigmaAO = 10;

% Interval length in consideration
T = 250;                                                                       % Large enough but finite constant

% Data Matrices
LB = 0.0; INC = 0.1; UB = 1.0;
arrPXi1 = LB:INC:UB;
size_pXi1 = numel(arrPXi1);
arrCCE = zeros(numCond,size_pXi1);

for CondBO = 1:numCond

    % Read from files taoA and taoO values derived from a Gaussian distribution
    fnametaoA = sprintf('Exp%dCond%d_Vec_taoA.csv',ExpR,CondBO);
    fnametaoO = sprintf('Exp%dCond%d_Vec_taoO.csv',ExpR,CondBO);
    Vec_taoA = dlmread(fnametaoA);
    Vec_taoO = dlmread(fnametaoO);

    indxPXi1 = size_pXi1 + 1;

    for PXi_1 = UB:-INC:LB

        PXi_0 = 1 - PXi_1;

        Vec_CCE = soa_InitMatrix(1,taoInstances);

        for indx_tao = 1:taoInstances

            % Do for each pair of taoA and taoO
            taoA = Vec_taoA(indx_tao);
            taoO = Vec_taoO(indx_tao);

            % Get the reported empricial baseline parameters
            [muA, sigmaA, muO, sigmaO] = soa_IBexperiment(ExpR, CondBO);

            % Compute CCE
            Z1 = sqrt(2*pi)*sigmaAO*T;
```

```
            Z0 = T^2;
            Theta = log((PXi_1*Z0)/(PXi_0*Z1));
            sigmaTot2 = sigmaA^2 + sigmaO^2 + sigmaAO^2;
            X = Theta - ((taoO-taoA-muAO)^2/(2*sigmaTot2)) + log(sigmaAO/sqrt(sigmaTot2));
            Vec_CCE(1,indx_tao) = (sqrt(sigmaTot2)/(2*pi*sigmaA*sigmaO*sigmaAO)) *...
                ( 1 / (1 + exp(-X)));

        end

        uCCE = mean(Vec_CCE(1,:)); sdCCE = std(Vec_CCE(1,:));
        fprintf('Condition %d\t P(Xi=1): %0.2f\n', CondBO, PXi_1);
        fprintf('CCE        :\t %0.2e(%0.2e)\n',  uCCE, sdCCE);

        indxPXi1 = indxPXi1 - 1;
        arrCCE(CondBO,indxPXi1) = uCCE;
    end
end

% Plot and store CCE as function of causal prior strength

soa_plotBehaviors(ExpR, arrCCE,  arrPXi1, fontsize, 1);
fnameCCEPXi = sprintf('Exp%d_CCEPXi.png',ExpR);
saveas(gcf,fnameCCEPXi);
```

```matlab
% Published: August 14, 2019
% Copyright
%   Lab for Neural Computation and Adaptation
%   RIKEN Center for Brain Science
%
% Objective: Compute the trial-to-trial Confidence on Causal Estimate (CCE)

% Cleaners
clear                                                                      % clears all variables from the Workspace
clc                                                                        % clears the Command Window
close all

% Graph display fonts
fontsize = 20;
sizeBin  = 200;

% Simulation Conditions
taoInstances = 35000;                                                      % Number of taoA and taoO instances to be generated
ExpR = 1; numCond = 3;                                                     % Experimental set-up
                                                                           %   Haggard et al. (2002): ExpR = 1; NumCond = 3; (Vol, Invol, Sham)
                                                                           %   Wolpe et al. (2013)  : ExpR = 2; NumCond = 3; (Low, Int, High)
tAp=0; dist_tAtO=250; tOp=tAp+dist_tAtO;                                   % Actual physical stimulus timings

% Optimal condition-independent parameters
muAO    = 230;
sigmaAO = 10;

% Interval length in consideration
T = 250;                                                                   % Large enough but finite constant

% Data Matrices
Vec_CCE  = soa_InitMatrix(numCond,taoInstances);
Vec_taoI = soa_InitMatrix(numCond,taoInstances);
Vec_Pc   = soa_InitMatrix(numCond,taoInstances);

for CondBO = 1:numCond

    % Simulated using the fitted P(Xi=1) optimal values

    if ExpR==1
        if CondBO == 1
            PXi_1 = 0.9;
        elseif CondBO == 2
            PXi_1 = 0.9;
        else
            PXi_1 = 0.1;
        end
    elseif ExpR==2
        if CondBO == 1
            PXi_1 = 0.9;
        elseif CondBO == 2
            PXi_1 = 0.6;
        else PXi_1 = 0.5;
        end
    end

    PXi_0 = 1 - PXi_1;

    % Read from files taoA and taoO values derived from a Gaussian distribution
    fnametaoA = sprintf('Exp%dCond%d_Vec_taoA.csv',ExpR,CondBO);
    fnametaoO = sprintf('Exp%dCond%d_Vec_taoO.csv',ExpR,CondBO);
    Vec_taoA = dlmread(fnametaoA);
    Vec_taoO = dlmread(fnametaoO);
```

```
    for indx_tao = 1:taoInstances

        % Do for each pair of taoA and taoO
        taoA = Vec_taoA(indx_tao);
        taoO = Vec_taoO(indx_tao);

        % Get the reported empricial baseline parameters
        [muA, sigmaA, muO, sigmaO] = soa_IBexperiment(ExpR, CondBO);

        % Compute CCE
        Z1 = sqrt(2*pi)*sigmaAO*T;
        Z0 = T^2;
        Theta = log((PXi_1*Z0)/(PXi_0*Z1));
        sigmaTot2 = sigmaA^2 + sigmaO^2 + sigmaAO^2;
        X = Theta - ((taoO-taoA-muAO)^2/(2*sigmaTot2)) + log(sigmaAO/sqrt(sigmaTot2));
        Vec_CCE(CondBO, indx_tao) = (sqrt(sigmaTot2)/(2*pi*sigmaA*sigmaO*sigmaAO)) *...
            ( 1 / (1 + exp(-X)));

        Vec_taoI(CondBO,indx_tao)  = taoO-taoA;

    end
end

% Plot and store trial-to-trial CCE as function of temporl disparity

sortedtaoI = Vec_taoI;
[sortedtaoI(1,:), sortIndx1] = sort(Vec_taoI(1,:));
[sortedtaoI(2,:), sortIndx2] = sort(Vec_taoI(2,:));
[sortedtaoI(3,:), sortIndx3] = sort(Vec_taoI(3,:));

sortedCCE = soa_sortMatrices(Vec_CCE, sortIndx1, sortIndx2, sortIndx3);
soa_plotErrorBars(ExpR, sortedtaoI, sortedCCE, fontsize, 1, sizeBin);
fnameCCE = sprintf('Exp%d_perTrialCCE.png',ExpR);
saveas(gcf,fnameCCE);
```

```
% Published: August 14, 2019
% Copyright
%   Lab for Neural Computation and Adaptation
%   RIKEN Center for Brain Science
%
% Objective: Return the means and standard deviations of the reported
%            baseline action and outcome timing judgment errors

function [mu_A, sigma_A, mu_O, sigma_O] = soa_IBexperiment(experiment_case, condition)

if experiment_case == 1
    % Haggard et al., 2002 (Nat Neurosci): Seminal intentional binding experiment
    %      Different keypress (i.e., the action) conditions

    if condition == 1
        mu_A = 6; sigma_A = 66;
    elseif condition == 2
        mu_A = 83; sigma_A = 83;
    elseif condition == 3
        mu_A = 32; sigma_A = 78;
    end
    mu_O = 15; sigma_O = 72;

elseif experiment_case == 2
    % Wolpe et al. 2013 (Exp Brain Res): Uncertainty is with the outcome
    %       Different tone (i.e., the outcome) conditions

    mu_A = -8; sigma_A = 75;
    if condition == 1
        mu_O = 35; sigma_O = 61;
    elseif condition == 2
        mu_O = 46; sigma_O = 66;
    elseif condition == 3
        mu_O = 95; sigma_O = 90;
    end
 end
```

```
% Published: August 14, 2019
% Copyright
%   Lab for Neural Computation and Adaptation
%   RIKEN Center for Brain Science
%
% Objective: Return the reported action and outcome perceptual shifts
%                for the operant conditions

function [trgtPrcShftA, trgtPrcShftO] = soa_IBTargets(experiment, condition)

if experiment == 1
    % Haggard et al., 2002 (Nat Neurosci): Seminal intentional binding experiment
    %      Different keypress (i.e., the action) conditions

    if condition == 1
        trgtPrcShftA =  15;
        trgtPrcShftO = -46;
    elseif condition == 2
        trgtPrcShftA = -27;
        trgtPrcShftO =  31;
    elseif condition == 3
        trgtPrcShftA = -7;
        trgtPrcShftO = -8;
    end

elseif experiment == 2
    % Wolpe et al. 2013 (Exp Brain Res): Uncertainty is with the outcome
    %       Different tone (i.e., the outcome) conditions

    if condition == 1
        trgtPrcShftA =  39;
        trgtPrcShftO = -51;
    elseif condition == 2
        trgtPrcShftA =  31;
        trgtPrcShftO = -65;
    elseif condition == 3
        trgtPrcShftA =   32;
        trgtPrcShftO = -105;
    end
end
```

```
% Published: August 14, 2019
% Copyright
%   Lab for Neural Computation and Adaptation
%   RIKEN Center for Brain Science
%
% Objective: Instantiate a rows-by-cols matrix with zero values

function [matrix_] = soa_InitMatrix(rows_, cols_)
    matrix_ = zeros(rows_, cols_);
```

```
% Published: August 14, 2019
% Copyright
%   Lab for Neural Computation and Adaptation
%   RIKEN Center for Brain Science
%
% Objective: Sort the cotents of the matrices

function [sortedMatrix] = soa_sortMatrices(vectMatrix, sortIndx1, sortIndx2, sortIndx3)

    sortedMatrix      = vectMatrix;
    sortedMatrix(1,:) = vectMatrix(1,sortIndx1);
    sortedMatrix(2,:) = vectMatrix(2,sortIndx2);
    sortedMatrix(3,:) = vectMatrix(3,sortIndx3);
```

```
% Published: August 14, 2019
% Copyright
%   Lab for Neural Computation and Adaptation
%   RIKEN Center for Brain Science
%
% Objective: Graph the action and outcome perceptual shifts as functions of
%       the strenth of the causal prior

function F = soa_plotPrcShts(experiment, arrPrcShftA, arrPrcShftO, arrPXi1, fontsize)

F = figure;
linewidth = 2;

if experiment == 1
    % Haggard et al., 2002 (Nat Neurosci): Seminal intentional binding experiment
    %      Different keypress (i.e., the action) conditions

    plot(arrPXi1,arrPrcShftA(1,:),'b-', arrPXi1,arrPrcShftO(1,:),'b--',...
         arrPXi1,arrPrcShftA(2,:),'r-', arrPXi1,arrPrcShftO(2,:),'r--',...
         arrPXi1,arrPrcShftA(3,:),'k-', arrPXi1,arrPrcShftO(3,:),'k--', 'Linewidth',linewidth);
    %legend('Voluntary Action', '    Tone', 'Involuntary MEP','    Tone','Sham TMS','    Tone', 'Location', 'northwest');
    lgnd = legend('Voluntary action',' and tone', 'Involuntary action',' and tone','Sham',' and tone', 'Location', 'northwest', 'Orientation','vertical');
    lgnd.FontSize = 18;
    set(lgnd.BoxFace, 'ColorType', 'truecoloralpha', 'ColorData', uint8(255*[1; 1; 1; 0.8]));

elseif experiment == 2
    % Wolpe et al. 2013 (Exp Brain Res): Uncertainty is with the outcome
    %       Different tone (i.e., the outcome) conditions

    hold on;
    plot(arrPXi1,arrPrcShftA(1,:),'Color', [0 0 250/255],       'LineStyle','-', 'Linewidth',linewidth);
    plot(arrPXi1,arrPrcShftO(1,:),'Color', [0 0 250/255],       'LineStyle','--','Linewidth',linewidth);
    plot(arrPXi1,arrPrcShftA(2,:),'Color', [0 140/255 255/255], 'LineStyle','-', 'Linewidth',linewidth);
    plot(arrPXi1,arrPrcShftO(2,:),'Color', [0 140/255 255/255], 'LineStyle','--','Linewidth',linewidth);
    plot(arrPXi1,arrPrcShftA(3,:),'Color', [0 240/255 255/255], 'LineStyle','-', 'Linewidth',linewidth);
    plot(arrPXi1,arrPrcShftO(3,:),'Color', [0 240/255 255/255], 'LineStyle','--','Linewidth',linewidth);
    lgnd = legend('Action', ' and low uncertainty tone', 'Action', ' and intermediate uncertainty tone', 'Action', ' and high uncertainty tone', 'Location', 'northwest');
    lgnd.FontSize = 18;
    set(lgnd.BoxFace, 'ColorType', 'truecoloralpha', 'ColorData', uint8(255*[1; 1; 1; 0.8]));
end

set(gca,'FontSize', fontsize);
set(gca, 'Box', 'on');
set(lgnd, 'Color', 'none');
set(gca,'color','white');
set(gca,'FontSize', fontsize);
```

```matlab
% Published: August 14, 2019
% Copyright
%   Lab for Neural Computation and Adaptation
%   RIKEN Center for Brain Science
%
% Objective: Plot the optimal bahaviors used in the figures of the paper with ERROR BARS displayed

function F = soa_plotErrorBars(experiment, arrAxes, arrBehavior, fontsize, flag, sizeBin)

F = figure;

markerSize = 27;
lineWidth = 0.1;

if experiment == 1
    % Haggard et al., 2002 (Nat Neurosci): Seminal intentional binding experiment
    %      Different keypress (i.e., the action) conditions

    hold all

    if flag == 1

        errorbar(mean(reshape(arrAxes(1,:),sizeBin,[]),1), mean(reshape(arrBehavior(1,:),sizeBin,[]),1), std(reshape(arrBehavior(1,:),sizeBin,[]),1),'b.', 'LineWidth',lineWidth,'MarkerSize',↙
markerSize);
        errorbar(mean(reshape(arrAxes(2,:),sizeBin,[]),1), mean(reshape(arrBehavior(2,:),sizeBin,[]),1), std(reshape(arrBehavior(2,:),sizeBin,[]),1),'r.', 'LineWidth',lineWidth,'MarkerSize',↙
markerSize);
        errorbar(mean(reshape(arrAxes(3,:),sizeBin,[]),1), mean(reshape(arrBehavior(3,:),sizeBin,[]),1), std(reshape(arrBehavior(3,:),sizeBin,[]),1),'k.', 'LineWidth',lineWidth,'MarkerSize',↙
markerSize);

        lgnd = legend('Voluntary condition','Involuntary condition','Sham condition', 'Location', 'northwest');

    elseif flag == 2
        errorbar(mean(reshape(arrAxes(2,:),sizeBin,[]),1), mean(reshape(arrBehavior(2,:),sizeBin,[]),1), std(reshape(arrBehavior(2,:),sizeBin,[]),1),'r.', 'LineWidth',lineWidth,'MarkerSize',↙
markerSize);
        errorbar(mean(reshape(arrAxes(1,:),sizeBin,[]),1), mean(reshape(arrBehavior(1,:),sizeBin,[]),1), std(reshape(arrBehavior(1,:),sizeBin,[]),1),'b.', 'LineWidth',lineWidth,'MarkerSize',↙
markerSize);

        lgnd = legend('Voluntary condition','Involuntary condition', 'Location', 'northwest');
    end

    set(lgnd.BoxFace, 'ColorType', 'truecoloralpha', 'ColorData', uint8(255*[1; 1; 1; 0.8]));
    set(gca, 'Box', 'on');
    hold off

elseif experiment == 2
    % Wolpe et al. 2013 (Exp Brain Res): Uncertainty is with the outcome
    %       Different tone (i.e., the outcome) conditions

    hold all

    if flag == 1

        errorbar(mean(reshape(arrAxes(1,:),sizeBin,[]),1), mean(reshape(arrBehavior(1,:),sizeBin,[]),1), std(reshape(arrBehavior(1,:),sizeBin,[]),1),'Color', [0 0 250/255],↙
'LineStyle','none','Marker', '.', 'LineWidth',lineWidth,'MarkerSize', markerSize);
        errorbar(mean(reshape(arrAxes(2,:),sizeBin,[]),1), mean(reshape(arrBehavior(2,:),sizeBin,[]),1), std(reshape(arrBehavior(2,:),sizeBin,[]),1),'Color', [0 140/255 255/255],↙
'LineStyle','none','Marker', '.', 'LineWidth',lineWidth,'MarkerSize',markerSize);
        errorbar(mean(reshape(arrAxes(3,:),sizeBin,[]),1), mean(reshape(arrBehavior(3,:),sizeBin,[]),1), std(reshape(arrBehavior(3,:),sizeBin,[]),1),'Color', [0 240/255 255/255],↙
'LineStyle','none','Marker', '.', 'LineWidth',lineWidth,'MarkerSize',markerSize);
    end

    lgnd = legend('Low uncertainty', 'Intermediate uncertainty', 'High uncertainty', 'Location', 'northwest');
    set(lgnd.BoxFace, 'ColorType', 'truecoloralpha', 'ColorData', uint8(255*[1; 1; 1; 0.8]));
    set(gca, 'Box', 'on');
    hold off
```

```
end
set(gca,'FontSize', fontsize);
```

```
% Function to graph the SoA related measures
% Added 09/06/2017

function F = soa_plotBehaviors(experiment, arrBehavior, arrPXi1, fontsize, behavior)

    F = figure;
    linewidth = 2;

    if experiment == 1

        if behavior == 1
            plot( arrPXi1,arrBehavior(1,:),'b', arrPXi1,arrBehavior(2,:),'r', arrPXi1,arrBehavior(3,:),'k', 'Linewidth',linewidth);
            %{
            hold all
            plot(arrPXi1,arrBehavior(1,:),'b','Linewidth',3);
            plot(arrPXi1,arrBehavior(2,:),'r','Linewidth',3);
            plot(arrPXi1,arrBehavior(3,:),'Color', [0 0 0]+0.05*13,'Linewidth',3);
            hold off
            %}
        elseif behavior == 3
            plot( arrPXi1,arrBehavior(2,:),'r', arrPXi1,arrBehavior(1,:),'b', arrPXi1,arrBehavior(3,:),'k', 'Linewidth',linewidth);
        elseif behavior == 0
            plot( arrPXi1,arrBehavior(1,:),'b', arrPXi1,arrBehavior(2,:),'r', 'Linewidth',linewidth);
        elseif behavior == 2
            ylim([0.0 1.0]);
            plot( arrPXi1,arrBehavior(1,:),'b', arrPXi1,arrBehavior(2,:),'r', 'Linewidth',linewidth);
        end

    elseif experiment == 2

        hold on;
        plot(arrPXi1,arrBehavior(1,:),'Color', [0 0 250/255],       'LineStyle','-','Linewidth',linewidth);
        plot(arrPXi1,arrBehavior(2,:),'Color', [0 140/255 255/255], 'LineStyle','-','Linewidth',linewidth);
        plot(arrPXi1,arrBehavior(3,:),'Color', [0 240/255 255/255], 'LineStyle','-','Linewidth',linewidth);
        hold off;

    elseif experiment == 3

        hold on;
        plot(arrPXi1,arrBehavior(1,:),'Color', [0 0 250/255],       'LineStyle','-','Linewidth',linewidth);
        plot(arrPXi1,arrBehavior(2,:),'Color', [0 140/255 255/255], 'LineStyle','-','Linewidth',linewidth);
        hold off;
    end

    %{
    xlabel('P(\xi=1) of Prior');
    if behavior == 1
        ylabel('Feeling of Agency');
    elseif behavior == 2
        ylabel('Judgment of Agency');
    elseif behavior == 3
        ylabel('Bias in Action Estimates');
    elseif behavior == 4
        ylabel('Bias in Outcome Estimates');
    end
    %}

    if experiment == 1

        if behavior == 1
            lgnd = legend('Voluntary condition','Involuntary condition','Sham condition', 'Location', 'northwest');
        elseif behavior ==3
            lgnd = legend('Voluntary condition','Involuntary condition','Sham condition', 'Location', 'northwest');
        else
```

```
            lgnd = legend('Voluntary condition','Involuntary condition','Sham condition', 'Location', 'northwest');
        end

    elseif experiment == 2

        lgnd = legend('Low uncertainty condition', 'Intermediate uncertainty condition', 'High uncertainty condition', 'Location', 'northeast');

    elseif experiment == 3

        lgnd = legend('Active, Instructed', 'Passive, Instructed', 'Location', 'northeast');
    end

    set(gca,'FontSize', fontsize);
    set(gca, 'Box', 'on');
    %set(lgnd, 'Color', 'none');
    set(gca,'color','white');
    set(lgnd.BoxFace, 'ColorType', 'truecoloralpha', 'ColorData', uint8(255*[1; 1; 1; 0.8]));


    % If you want to bold
    %ylabel('Feeling of Agency', 'FontWeight', 'bold');
```
